# Supplementary material for: Peritonsillar abscess may not always be a complication of acute tonsillitis: A prospective cohort study
Source: PLoS One. 2020 Apr 3;15(4):e0228122. doi: 10.1371/journal.pone.0228122 (PMC7122714; doi:10.1371/journal.pone.0228122)
Supplement: S2 File — (DOCX) [file pone.0228122.s002.docx]

Explanations:

Group: AT=1, PT= 2, PTA =3

Age: years

Gender: 1= male, 2= female

Smoking: 1= yes, 2= no, 3= quit

Alcohol= 2= no, 1 = yes

Alcohol, overuse: 2= no, 1= yes

Antibiotic: 1= yes, 2= no

Antibiotic2: antibiotic treatment more than 24 h

Duration of symptoms: days

Duration of symptoms (group): 1= 1-3d 2= >4d

Unilateral: 1= yes, 2= no

Common cold: 1= yes, 2= no

Fever: 1= yes, 2= no

Earlier tonsillar infections: 1= AT, 2= CT, 3= PTA , 4= nothing

Oral hygiene: 1= good 2= poor

Tonsillar findings: 1= erythema, 2= exudates, 3= No findings 4= erythema + exudates

Bacterial findings: 1= normal flora 2= Streptococcus pyogenes 3= Streptococcus angiosus 4= Fusobacterium necrophorum

Amyl: U/l

CRP: mg/ml

Bilateral PTA: 1= yes, 2= no
